# Supplementary material for: A Proof-of-Concept Protein Microarray-Based Approach for Serotyping of Salmonella enterica Strains
Source: Pathogens. 2024 Apr 25;13(5):355. doi: 10.3390/pathogens13050355 (PMC11124431; doi:10.3390/pathogens13050355)
Supplement: Supplementary file 1 [file pathogens-13-00355-s001.zip › Supplementary File S1.pdf]

For Investigational Use Only. Not Intended for Use in Clinical Diagnostics.

**Operator**	---
**Sample ID**	---
**Experiment ID**	S.e.e._Budapest
**Date of Result**	Thu May 31 18:06:40 2012
**Assay Name**	salm-pm1
**Assay ID**	10624
**Well Position**	00 (00-H)
**Software Version**	0.4
**Device**	---
**biotin**	positive
**negative control**	negative
**invA**	positive
**galF**	positive
**manC**	positive

score	spp	serovare	strain	group O			H1	H2
0.0 (100.0 %)	I	Budapest	CDC23	B (O:4)	4	1,4,12,[27]	g,t	-
2.74 (98.93 %)	I	Agona	CDC1636	B (O:4)	4	1,4,[5],12	f,g,s	[1,2]

Resistance Genotyping

description	result
aminoglycoside resistance (aac, aad, ant, arm)	negative
beta lactam resistance (dha, ctxM, pse, aac, blaCNY, oxa, cmy, per, shv, tem)	negative
bleomycin resistance (ble)	negative
chloramphenicol resistance (cat, cml)	negative
erythomycin and roxythromycin resistance (qnr)	negative
erythromycin resistance (ere)	negative
florfenicol and chloramphenicol resistance (floR)	negative
fluoroquinolone resistance (qnr)	negative
gentamicin resistance (aac)	negative
imipenem resistance (kpc)	negative
kanamycin resistance (aph)	negative
quinolone resistance (qnr)	negative
streptomycin resistance (sph, Str)	negative
sulfonamide resistance (sul)	negative
tetracycline resistance (tet)	negative
trimethoprim resistance (dfr)	negative

Virulences

description	result
heat-stable enterotoxin (astA)	negative
integrases (intI)	negative
plasmid Vi (tviA)	negative

For Investigational Use Only. Not Intended for Use in Clinical Diagnostics.

**Operator**	---
**Sample ID**	---
**Experiment ID**	S.e.e. Choleraeusis
**Date of Result**	Thu May 31 18:05:40 2012
**Assay Name**	salm-pm1
**Assay ID**	10624
**Well Position**	00 (00-H)
**Software Version**	0.4
**Device**	---
**biotin**	positive
**negative control**	negative
**invA**	negative
**galF**	positive
**manC**	positive

score	spp	serovare	strain	group O			H1	H2
0.0 (100.0 %)	I	Choleraesuis	CDC34	C1 (O:7)	7	6,7	c	1,5
4.05 (98.41 %)	I	Choleraesuis	DSM14846	C1 (O:7)	7	6,7	c	1,5

Resistance Genotyping

description	result
aminoglycoside resistance (aac, aad, ant, arm)	negative
beta lactam resistance (dha, ctxM, pse, aac, blaCNY, oxa, cmy, per, shv, tem)	negative
bleomycin resistance (ble)	negative
chloramphenicol resistance (cat, cml)	negative
erythomycin and roxythromycin resistance (qnr)	negative
erythromycin resistance (ere)	negative
florfenicol and chloramphenicol resistance (floR)	negative
fluoroquinolone resistance (qnr)	negative
gentamicin resistance (aac)	negative
imipenem resistance (kpc)	negative
kanamycin resistance (aph)	negative
quinolone resistance (qnr)	negative
streptomycin resistance (sph, Str)	negative
sulfonamide resistance (sul)	negative
tetracycline resistance (tet)	negative
trimethoprim resistance (dfr)	negative

Virulences

description	result
heat-stable enterotoxin (astA)	negative
integrases (intI)	negative
plasmid Vi (tviA)	negative

For Investigational Use Only. Not Intended for Use in Clinical Diagnostics.

**Operator**	---
**Sample ID**	---
**Experiment ID**	S.e.e._Moscow
**Date of Result**	Thu May 31 18:12:56 2012
**Assay Name**	salm-pm1
**Assay ID**	10624
**Well Position**	00 (00-H)
**Software Version**	0.4
**Device**	---
**biotin**	positive
**negative control**	negative
**invA**	positive
**galF**	positive
**manC**	positive

score	spp	serovare	strain	group O			H1	H2
0.27 (99.89 %)	I	Moscow	CDC67	D1 (O:9)	9	1,9,12	g,q	-
3.41 (98.66 %)	I	Naestved	SGSC3612	D1 (O:9)	9	1,9,12	g,p,s	-

Resistance Genotyping

description	result
aminoglycoside resistance (aac, aad, ant, arm)	negative
beta lactam resistance (dha, ctxM, pse, aac, blaCNY, oxa, cmy, per, shv, tem)	negative
bleomycin resistance (ble)	negative
chloramphenicol resistance (cat, cml)	negative
erythomycin and roxythromycin resistance (qnr)	negative
erythromycin resistance (ere)	negative
florfenicol and chloramphenicol resistance (floR)	negative
fluoroquinolone resistance (qnr)	negative
gentamicin resistance (aac)	negative
imipenem resistance (kpc)	negative
kanamycin resistance (aph)	negative
quinolone resistance (qnr)	negative
streptomycin resistance (sph, Str)	negative
sulfonamide resistance (sul)	negative
tetracycline resistance (tet)	negative
trimethoprim resistance (dfr)	negative

Virulences

description	result
heat-stable enterotoxin (astA)	negative
integrases (intI)	negative
plasmid Vi (tviA)	negative

For Investigational Use Only. Not Intended for Use in Clinical Diagnostics.

**Operator**	---
**Sample ID**	---
**Experiment ID**	S.e.e._Saintpaul
**Date of Result**	Thu May 31 10:00:29 2012
**Assay Name**	salm-pm1
**Assay ID**	10624
**Well Position**	00 (00-H)
**Software Version**	0.4
**Device**	---
**biotin**	positive
**negative control**	negative
**invA**	positive
**galF**	positive
**manC**	positive

score	spp	serovare	strain	group O			H1	H2
2.06 (99.19 %)	I	Saintpaul	CDC108	B (O:4)	4	1,4,[5],12	e,h	1,2
6.81 (97.33 %)	I	Bredeney	CDC112	B (O:4)	4	1,4,12,27	l,v	1,7

Resistance Genotyping

description	result
aminoglycoside resistance (aac, aad, ant, arm)	negative
beta lactam resistance (dha, ctxM, pse, aac, blaCNY, oxa, cmy, per, shv, tem)	negative
bleomycin resistance (ble)	negative
chloramphenicol resistance (cat, cml)	negative
erythomycin and roxythromycin resistance (qnr)	negative
erythromycin resistance (ere)	negative
florfenicol and chloramphenicol resistance (floR)	negative
fluoroquinolone resistance (qnr)	negative
gentamicin resistance (aac)	negative
imipenem resistance (kpc)	negative
kanamycin resistance (aph)	negative
quinolone resistance (qnr)	negative
streptomycin resistance (sph, Str)	negative
sulfonamide resistance (sul)	negative
tetracycline resistance (tet)	negative
trimethoprim resistance (dfr)	negative

Virulences

description	result
heat-stable enterotoxin (astA)	negative
integrases (intI)	negative
plasmid Vi (tviA)	negative

For Investigational Use Only. Not Intended for Use in Clinical Diagnostics.

**Operator**	---
**Sample ID**	---
**Experiment ID**	S.e.e._Mississippi
**Date of Result**	Thu May 31 18:15:35 2012
**Assay Name**	salm-pm1
**Assay ID**	10624
**Well Position**	00 (00-H)
**Software Version**	0.4
**Device**	---
**biotin**	positive
**negative control**	negative
**invA**	positive
**galF**	positive
**manC**	positive

score	spp	serovare	strain	group O			H1	H2
0.0 (100.0 %)	I	Mississippi	CDC154	G (O:13)	13	1,13,23	b	1,5
3.32 (98.7 %)	I	Mississippi	CDC154	G (O:13)	13	1,13,23	b	1,5

Resistance Genotyping

description	result
aminoglycoside resistance (aac, aad, ant, arm)	negative
beta lactam resistance (dha, ctxM, pse, aac, blaCNY, oxa, cmy, per, shv, tem)	negative
bleomycin resistance (ble)	negative
chloramphenicol resistance (cat, cml)	negative
erythomycin and roxythromycin resistance (qnr)	negative
erythromycin resistance (ere)	negative
florfenicol and chloramphenicol resistance (floR)	negative
fluoroquinolone resistance (qnr)	negative
gentamicin resistance (aac)	negative
imipenem resistance (kpc)	negative
kanamycin resistance (aph)	negative
quinolone resistance (qnr)	negative
streptomycin resistance (sph, Str)	negative
sulfonamide resistance (sul)	negative
tetracycline resistance (tet)	negative
trimethoprim resistance (dfr)	negative

Virulences

description	result
heat-stable enterotoxin (astA)	negative
integrases (intI)	negative
plasmid Vi (tviA)	negative

For Investigational Use Only. Not Intended for Use in Clinical Diagnostics.

**Operator**	---
**Sample ID**	---
**Experiment ID**	S.e.e._Cubana
**Date of Result**	Thu May 31 10:06:05 2012
**Assay Name**	salm-pm1
**Assay ID**	10624
**Well Position**	00 (00-H)
**Software Version**	0.4
**Device**	---
**biotin**	positive
**negative control**	negative
**invA**	positive
**galF**	positive
**manC**	positive

score	spp	serovare	strain	group O			H1	H2
0.61 (99.76 %)	I	Cubana	CDC207	G (O:13)	13	1,13,23	z29	-
5.31 (97.92 %)	I	Ajiobo	CDC527	G (O:13)	13	13,23	z4,z23	-

Resistance Genotyping

description	result
aminoglycoside resistance (aac, aad, ant, arm)	negative
beta lactam resistance (dha, ctxM, pse, aac, blaCNY, oxa, cmy, per, shv, tem)	negative
bleomycin resistance (ble)	negative
chloramphenicol resistance (cat, cml)	negative
erythomycin and roxythromycin resistance (qnr)	negative
erythromycin resistance (ere)	negative
florfenicol and chloramphenicol resistance (floR)	negative
fluoroquinolone resistance (qnr)	negative
gentamicin resistance (aac)	negative
imipenem resistance (kpc)	negative
kanamycin resistance (aph)	negative
quinolone resistance (qnr)	negative
streptomycin resistance (sph, Str)	negative
sulfonamide resistance (sul)	negative
tetracycline resistance (tet)	negative
trimethoprim resistance (dfr)	negative

Virulences

description	result
heat-stable enterotoxin (astA)	negative
integrases (intI)	negative
plasmid Vi (tviA)	negative

For Investigational Use Only. Not Intended for Use in Clinical Diagnostics.

**Operator**	---
**Sample ID**	---
**Experiment ID**	S.e.e._Stanleyville
**Date of Result**	Thu May 31 18:15:56 2012
**Assay Name**	salm-pm1
**Assay ID**	10624
**Well Position**	00 (00-H)
**Software Version**	0.4
**Device**	---
**biotin**	positive
**negative control**	negative
**invA**	positive
**galF**	positive
**manC**	positive

score	spp	serovare	strain	group O			H1	H2
0.0 (100.0 %)	I	Stanleyville	CDC223	B (O:4)	4	1,4,[5],12,[27]	z4,z23	[1,2]
9.61 (96.23 %)	I	Derby	K007	B (O:4)	4	1,4,[5],12	f,g	[1,2]

Resistance Genotyping

description	result
aminoglycoside resistance (aac, aad, ant, arm)	negative
beta lactam resistance (dha, ctxM, pse, aac, blaCNY, oxa, cmy, per, shv, tem)	negative
bleomycin resistance (ble)	negative
chloramphenicol resistance (cat, cml)	negative
erythomycin and roxythromycin resistance (qnr)	negative
erythromycin resistance (ere)	negative
florfenicol and chloramphenicol resistance (floR)	negative
fluoroquinolone resistance (qnr)	negative
gentamicin resistance (aac)	negative
imipenem resistance (kpc)	negative
kanamycin resistance (aph)	negative
quinolone resistance (qnr)	negative
streptomycin resistance (sph, Str)	negative
sulfonamide resistance (sul)	negative
tetracycline resistance (tet)	negative
trimethoprim resistance (dfr)	negative

Virulences

description	result
heat-stable enterotoxin (astA)	negative
integrases (intI)	negative
plasmid Vi (tviA)	negative

For Investigational Use Only. Not Intended for Use in Clinical Diagnostics.

**Operator**	---
**Sample ID**	---
**Experiment ID**	S.e.e. Gloucester
**Date of Result**	Thu May 31 18:17:42 2012
**Assay Name**	salm-pm1
**Assay ID**	10624
**Well Position**	00 (00-H)
**Software Version**	0.4
**Device**	---
**biotin**	positive
**negative control**	negative
**invA**	positive
**galF**	positive
**manC**	positive

score	spp	serovare	strain	group O			H1	H2
0.0 (100.0 %)	I	Gloucester	CDC443	B (O:4)	4	1,4,12,27	i	l,w
6.08 (97.62 %)	I	1,4,[5],12:i:- (Typhimurium monophasic)	CDCQA126	B (O:4)	4	1,4,[5],12	i	-

Resistance Genotyping

description	result
aminoglycoside resistance (aac, aad, ant, arm)	negative
beta lactam resistance (dha, ctxM, pse, aac, blaCNY, oxa, cmy, per, shv, tem)	negative
bleomycin resistance (ble)	negative
chloramphenicol resistance (cat, cml)	negative
erythomycin and roxythromycin resistance (qnr)	negative
erythromycin resistance (ere)	negative
florfenicol and chloramphenicol resistance (floR)	negative
fluoroquinolone resistance (qnr)	negative
gentamicin resistance (aac)	negative
imipenem resistance (kpc)	negative
kanamycin resistance (aph)	negative
quinolone resistance (qnr)	negative
streptomycin resistance (sph, Str)	negative
sulfonamide resistance (sul)	negative
tetracycline resistance (tet)	negative
trimethoprim resistance (dfr)	negative

Virulences

description	result
heat-stable enterotoxin (astA)	negative
integrases (intI)	negative
plasmid Vi (tviA)	negative

For Investigational Use Only. Not Intended for Use in Clinical Diagnostics.

**Operator**	---
**Sample ID**	---
**Experiment ID**	S.e.e._Agama
**Date of Result**	Thu May 31 18:16:02 2012
**Assay Name**	salm-pm1
**Assay ID**	10624
**Well Position**	00 (00-H)
**Software Version**	0.4
**Device**	---
**biotin**	positive
**negative control**	negative
**invA**	positive
**galF**	positive
**manC**	positive

score	spp	serovare	strain	group O			H1	H2
0.0 (100.0 %)	I	Agama	CDC513	B (O:4)	4	4,12	i	1,6
6.92 (97.29 %)	I	Typhimurium	LT2	B (O:4)	4	1,4,[5],12	i	1,2

Resistance Genotyping

description	result
aminoglycoside resistance (aac, aad, ant, arm)	negative
beta lactam resistance (dha, ctxM, pse, aac, blaCNY, oxa, cmy, per, shv, tem)	negative
bleomycin resistance (ble)	negative
chloramphenicol resistance (cat, cml)	negative
erythomycin and roxythromycin resistance (qnr)	negative
erythromycin resistance (ere)	negative
florfenicol and chloramphenicol resistance (floR)	negative
fluoroquinolone resistance (qnr)	negative
gentamicin resistance (aac)	negative
imipenem resistance (kpc)	negative
kanamycin resistance (aph)	negative
quinolone resistance (qnr)	negative
streptomycin resistance (sph, Str)	negative
sulfonamide resistance (sul)	negative
tetracycline resistance (tet)	negative
trimethoprim resistance (dfr)	negative

Virulences

description	result
heat-stable enterotoxin (astA)	negative
integrases (intI)	negative
plasmid Vi (tviA)	negative

For Investigational Use Only. Not Intended for Use in Clinical Diagnostics.

Operator ---
Sample ID ---
Experiment ID S.e.e._Goeteborg
Date of Result Thu May 31 09:54:43 2012
Assay Name salm-pm1
Assay ID 10624
Well Position 00 (00-H)
Software Version 0.4
Device ---
biotin positive
negative control negative
invA negative
galF positive
manC positive

score	spp	serovare	strain	group O			H1	H2
4.76 (98.13 %)	I	Goeteborg	CDC696	D1 (O:9)	9	9,12	c	1,5
10.38 (95.93 %)	I	Panama	CDC73	D1 (O:9)	9	1,9,12	l,v	1,5

Resistance Genotyping

description	result
aminoglycoside resistance (aac, aad, ant, arm)	negative
beta lactam resistance (dha, ctxM, pse, aac, blaCNY, oxa, cmy, per, shv, tem)	negative
bleomycin resistance (ble)	negative
chloramphenicol resistance (cat, cml)	negative
erythomycin and roxythromycin resistance (qnr)	negative
erythromycin resistance (ere)	negative
florfenicol and chloramphenicol resistance (floR)	negative
fluoroquinolone resistance (qnr)	negative
gentamicin resistance (aac)	negative
imipenem resistance (kpc)	negative
kanamycin resistance (aph)	negative
quinolone resistance (qnr)	negative
streptomycin resistance (sph, Str)	negative
sulfonamide resistance (sul)	negative
tetracycline resistance (tet)	negative
trimethoprim resistance (dfr)	negative

Virulences

description	result
heat-stable enterotoxin (astA)	negative
integrases (intI)	negative
plasmid Vi (tviA)	negative

For Investigational Use Only. Not Intended for Use in Clinical Diagnostics.

**Operator**	---
**Sample ID**	---
**Experiment ID**	S.e.e._Potsdam
**Date of Result**	Thu May 31 10:05:26 2012
**Assay Name**	salm-pm1
**Assay ID**	10624
**Well Position**	00 (00-H)
**Software Version**	0.4
**Device**	---
**biotin**	positive
**negative control**	negative
**invA**	positive
**galF**	positive
**manC**	positive

score	spp	serovare	strain	group O			H1	H2
1.04 (99.59 %)	I	Potsdam	CDC876	C1 (O:7)	7	6,7,14	l,v	e,n,z15
4.58 (98.2 %)	I	Kenya	CDC497	C1 (O:7)	7	6,7	l,z13	e,n,x

Resistance Genotyping

description	result
aminoglycoside resistance (aac, aad, ant, arm)	negative
beta lactam resistance (dha, ctxM, pse, aac, blaCNY, oxa, cmy, per, shv, tem)	negative
bleomycin resistance (ble)	negative
chloramphenicol resistance (cat, cml)	negative
erythomycin and roxythromycin resistance (qnr)	negative
erythromycin resistance (ere)	negative
florfenicol and chloramphenicol resistance (floR)	negative
fluoroquinolone resistance (qnr)	negative
gentamicin resistance (aac)	negative
imipenem resistance (kpc)	negative
kanamycin resistance (aph)	negative
quinolone resistance (qnr)	negative
streptomycin resistance (sph, Str)	negative
sulfonamide resistance (sul)	negative
tetracycline resistance (tet)	negative
trimethoprim resistance (dfr)	negative

Virulences

description	result
heat-stable enterotoxin (astA)	negative
integrases (intI)	negative
plasmid Vi (tviA)	negative

For Investigational Use Only. Not Intended for Use in Clinical Diagnostics.

**Operator**	---
**Sample ID**	---
**Experiment ID**	S.e.e. Dublin
**Date of Result**	Thu May 31 18:05:34 2012
**Assay Name**	salm-pm1
**Assay ID**	10624
**Well Position**	00 (00-H)
**Software Version**	0.4
**Device**	---
**biotin**	positive
**negative control**	negative
**invA**	positive
**galF**	positive
**manC**	positive

score	spp	serovare	strain	group O			H1	H2
0.34 (99.86 %)	I	Dublin	CDC65	D1 (O:9)	9	1,9,12[Vi]	g,p	-
2.25 (99.12 %)	I	Naestved	SGSC3612	D1 (O:9)	9	1,9,12	g,p,s	-

Resistance Genotyping

description	result
aminoglycoside resistance (aac, aad, ant, arm)	negative
beta lactam resistance (dha, ctxM, pse, aac, blaCNY, oxa, cmy, per, shv, tem)	negative
bleomycin resistance (ble)	negative
chloramphenicol resistance (cat, cml)	negative
erythomycin and roxythromycin resistance (qnr)	negative
erythromycin resistance (ere)	negative
florfenicol and chloramphenicol resistance (floR)	negative
fluoroquinolone resistance (qnr)	negative
gentamicin resistance (aac)	negative
imipenem resistance (kpc)	negative
kanamycin resistance (aph)	negative
quinolone resistance (qnr)	negative
streptomycin resistance (sph, Str)	negative
sulfonamide resistance (sul)	negative
tetracycline resistance (tet)	negative
trimethoprim resistance (dfr)	negative

Virulences

description	result
heat-stable enterotoxin (astA)	negative
integrases (intI)	negative
plasmid Vi (tviA)	negative

For Investigational Use Only. Not Intended for Use in Clinical Diagnostics.

**Operator**	---
**Sample ID**	---
**Experiment ID**	CDC68_S.e.e._Blegdam
**Date of Result**	Thu May 31 10:09:39 2012
**Assay Name**	salm-pm1
**Assay ID**	10624
**Well Position**	00 (00-H)
**Software Version**	0.4
**Device**	---
**biotin**	positive
**negative control**	negative
**invA**	negative
**galF**	positive
**manC**	positive

score	spp	serovare	strain	group O			H1	H2
2.06 (99.19 %)	I	Blegdam	CDC68	D1 (O:9)	9	9,12	g,m,q	-
4.53 (98.22 %)	I	Blegdam	CDC090361	D1 (O:9)	9	9,12	g,m,q	-

Resistance Genotyping

description	result
aminoglycoside resistance (aac, aad, ant, arm)	negative
beta lactam resistance (dha, ctxM, pse, aac, blaCNY, oxa, cmy, per, shv, tem)	negative
bleomycin resistance (ble)	negative
chloramphenicol resistance (cat, cml)	negative
erythomycin and roxythromycin resistance (qnr)	negative
erythromycin resistance (ere)	negative
florfenicol and chloramphenicol resistance (floR)	negative
fluoroquinolone resistance (qnr)	negative
gentamicin resistance (aac)	negative
imipenem resistance (kpc)	negative
kanamycin resistance (aph)	negative
quinolone resistance (qnr)	negative
streptomycin resistance (sph, Str)	negative
sulfonamide resistance (sul)	negative
tetracycline resistance (tet)	negative
trimethoprim resistance (dfr)	negative

Virulences

description	result
heat-stable enterotoxin (astA)	negative
integrases (intI)	negative
plasmid Vi (tviA)	negative

For Investigational Use Only. Not Intended for Use in Clinical Diagnostics.

**Operator**	---
**Sample ID**	---
**Experiment ID**	S.e.e._Inverness
**Date of Result**	Thu May 31 10:06:49 2012
**Assay Name**	salm-pm1
**Assay ID**	10624
**Well Position**	00 (00-H)
**Software Version**	0.4
**Device**	---
**biotin**	positive
**negative control**	negative
**invA**	positive
**galF**	positive
**manC**	positive

score	spp	serovare	strain	group O			H1	H2
0.9 (99.65 %)	I	Inverness	CDC171	P (O:38)	38	38	k	1,6
6.72 (97.36 %)	I	Gera	CDC1316	T (O:42)	42	1,42	z4,z23	1,6

Resistance Genotyping

description	result
aminoglycoside resistance (aac, aad, ant, arm)	negative
beta lactam resistance (dha, ctxM, pse, aac, blaCNY, oxa, cmy, per, shv, tem)	negative
bleomycin resistance (ble)	negative
chloramphenicol resistance (cat, cml)	negative
erythomycin and roxythromycin resistance (qnr)	negative
erythromycin resistance (ere)	negative
florfenicol and chloramphenicol resistance (floR)	negative
fluoroquinolone resistance (qnr)	negative
gentamicin resistance (aac)	negative
imipenem resistance (kpc)	negative
kanamycin resistance (aph)	negative
quinolone resistance (qnr)	negative
streptomycin resistance (sph, Str)	negative
sulfonamide resistance (sul)	negative
tetracycline resistance (tet)	negative
trimethoprim resistance (dfr)	negative

Virulences

description	result
heat-stable enterotoxin (astA)	negative
integrases (intI)	negative
plasmid Vi (tviA)	negative

For Investigational Use Only. Not Intended for Use in Clinical Diagnostics.

**Operator**	---
**Sample ID**	---
**Experiment ID**	S.e.e._California
**Date of Result**	Thu May 31 10:09:55 2012
**Assay Name**	salm-pm1
**Assay ID**	10624
**Well Position**	00 (00-H)
**Software Version**	0.4
**Device**	---
**biotin**	positive
**negative control**	negative
**invA**	positive
**galF**	positive
**manC**	positive

score	spp	serovare	strain	group O			H1	H2
0.65 (99.75 %)	I	California	CDC1109	B (O:4)	4	4,12	g,m,t	[z67]
7.37 (97.11 %)	I	Agona	CDC1636	B (O:4)	4	1,4,[5],12	f,g,s	[1,2]

Resistance Genotyping

description	result
aminoglycoside resistance (aac, aad, ant, arm)	negative
beta lactam resistance (dha, ctxM, pse, aac, blaCNY, oxa, cmy, per, shv, tem)	negative
bleomycin resistance (ble)	negative
chloramphenicol resistance (cat, cml)	negative
erythomycin and roxythromycin resistance (qnr)	negative
erythromycin resistance (ere)	negative
florfenicol and chloramphenicol resistance (floR)	negative
fluoroquinolone resistance (qnr)	negative
gentamicin resistance (aac)	negative
imipenem resistance (kpc)	negative
kanamycin resistance (aph)	negative
quinolone resistance (qnr)	negative
streptomycin resistance (sph, Str)	negative
sulfonamide resistance (sul)	negative
tetracycline resistance (tet)	negative
trimethoprim resistance (dfr)	negative

Virulences

description	result
heat-stable enterotoxin (astA)	negative
integrases (intI)	negative
plasmid Vi (tviA)	negative

For Investigational Use Only. Not Intended for Use in Clinical Diagnostics.

**Operator**	---
**Sample ID**	---
**Experiment ID**	S.e.e._Nitra
**Date of Result**	Thu May 31 10:09:34 2012
**Assay Name**	salm-pm1
**Assay ID**	10624
**Well Position**	00 (00-H)
**Software Version**	0.4
**Device**	---
**biotin**	positive
**negative control**	negative
**invA**	positive
**galF**	positive
**manC**	positive

score	spp	serovare	strain	group O			H1	H2
0.89 (99.65 %)	I	Nitra	CDC1280	A (O:2)	2	2,12	g,m	-
1.89 (99.26 %)	I	Enteritidis	DSM14221	D1 (O:9)	9	1,9,12	g,m	-

Resistance Genotyping

description	result
aminoglycoside resistance (aac, aad, ant, arm)	negative
beta lactam resistance (dha, ctxM, pse, aac, blaCNY, oxa, cmy, per, shv, tem)	negative
bleomycin resistance (ble)	negative
chloramphenicol resistance (cat, cml)	negative
erythomycin and roxythromycin resistance (qnr)	negative
erythromycin resistance (ere)	negative
florfenicol and chloramphenicol resistance (floR)	negative
fluoroquinolone resistance (qnr)	negative
gentamicin resistance (aac)	negative
imipenem resistance (kpc)	negative
kanamycin resistance (aph)	negative
quinolone resistance (qnr)	negative
streptomycin resistance (sph, Str)	negative
sulfonamide resistance (sul)	negative
tetracycline resistance (tet)	negative
trimethoprim resistance (dfr)	negative

Virulences

description	result
heat-stable enterotoxin (astA)	negative
integrases (intI)	negative
plasmid Vi (tviA)	negative

For Investigational Use Only. Not Intended for Use in Clinical Diagnostics.

Operator ---

Sample ID ---

Experiment ID S.e.e._Uno

Date of Result Thu May 31 10:08:29 2012

Assay Name salm-pm1

Assay ID 10624

Well Position 00 (00-H)

Software Version 0.4

Device ---

biotin positive

negative control negative

invA positive

galF positive

manC positive

score	spp	serovare	strain	group O			H1	H2
0.86 (99.66 %)	I	Uno	CDC1697	C2-C3 (O:8)	8	6,8	z29	[e,n,z15]
6.94 (97.28 %)	I	Hadar	CDC347	C2-C3 (O:8)	8	6,8	z10	e,n,x

Resistance Genotyping

description	result
aminoglycoside resistance (aac, aad, ant, arm)	negative
beta lactam resistance (dha, ctxM, pse, aac, blaCNY, oxa, cmy, per, shv, tem)	negative
bleomycin resistance (ble)	negative
chloramphenicol resistance (cat, cml)	negative
erythomycin and roxythromycin resistance (qnr)	negative
erythromycin resistance (ere)	negative
florfenicol and chloramphenicol resistance (floR)	negative
fluoroquinolone resistance (qnr)	negative
gentamicin resistance (aac)	negative
imipenem resistance (kpc)	negative
kanamycin resistance (aph)	negative
quinolone resistance (qnr)	negative
streptomycin resistance (sph, Str)	negative
sulfonamide resistance (sul)	negative
tetracycline resistance (tet)	negative
trimethoprim resistance (dfr)	negative

Virulences

description	result
heat-stable enterotoxin (astA)	negative
integrases (intI)	negative
plasmid Vi (tviA)	negative

For Investigational Use Only. Not Intended for Use in Clinical Diagnostics.

**Operator**	---
**Sample ID**	---
**Experiment ID**	S.e.e._Breukelen
**Date of Result**	Thu May 31 10:08:34 2012
**Assay Name**	salm-pm1
**Assay ID**	10624
**Well Position**	00 (00-H)
**Software Version**	0.4
**Device**	---
**biotin**	positive
**negative control**	negative
**invA**	positive
**galF**	positive
**manC**	positive

score	spp	serovare	strain	group O			H1	H2
1.15 (99.55 %)	I	Breukelen	CDC1699	C2-C3 (O:8)	8	6,8	l,z13,[z28]	e,n,z15
11.36 (95.54 %)	I	Hadar	CDC347	C2-C3 (O:8)	8	6,8	z10	e,n,x

Resistance Genotyping

description	result
aminoglycoside resistance (aac, aad, ant, arm)	negative
beta lactam resistance (dha, ctxM, pse, aac, blaCNY, oxa, cmy, per, shv, tem)	negative
bleomycin resistance (ble)	negative
chloramphenicol resistance (cat, cml)	negative
erythomycin and roxythromycin resistance (qnr)	negative
erythromycin resistance (ere)	negative
florfenicol and chloramphenicol resistance (floR)	negative
fluoroquinolone resistance (qnr)	negative
gentamicin resistance (aac)	negative
imipenem resistance (kpc)	negative
kanamycin resistance (aph)	negative
quinolone resistance (qnr)	negative
streptomycin resistance (sph, Str)	negative
sulfonamide resistance (sul)	negative
tetracycline resistance (tet)	negative
trimethoprim resistance (dfr)	negative

Virulences

description	result
heat-stable enterotoxin (astA)	negative
integrases (intI)	negative
plasmid Vi (tviA)	negative

For Investigational Use Only. Not Intended for Use in Clinical Diagnostics.

**Operator**	---
**Sample ID**	---
**Experiment ID**	S.e.e._Corvallis
**Date of Result**	Thu May 31 10:08:23 2012
**Assay Name**	salm-pm1
**Assay ID**	10624
**Well Position**	00 (00-H)
**Software Version**	0.4
**Device**	---
**biotin**	positive
**negative control**	negative
**invA**	positive
**galF**	positive
**manC**	positive

score	spp	serovare	strain	group O			H1	H2
1.0 (99.61 %)	I	Corvallis	CDC1770	C2-C3 (O:8)	8	8,20	z4,z23	[z6]
7.8 (96.94 %)	I	Kentucky	CDC2590	C2-C3 (O:8)	8	8,20	i	z6

Resistance Genotyping

description	result
aminoglycoside resistance (aac, aad, ant, arm)	positive
beta lactam resistance (dha, ctxM, pse, aac, blaCNY, oxa, cmy, per, shv, tem)	negative
bleomycin resistance (ble)	negative
chloramphenicol resistance (cat, cml)	positive
erythomycin and roxythromycin resistance (qnr)	negative
erythromycin resistance (ere)	negative
florfenicol and chloramphenicol resistance (floR)	negative
fluoroquinolone resistance (qnr)	negative
gentamicin resistance (aac)	negative
imipenem resistance (kpc)	negative
kanamycin resistance (aph)	negative
quinolone resistance (qnr)	negative
streptomycin resistance (sph, Str)	negative
sulfonamide resistance (sul)	positive
tetracycline resistance (tet)	negative
trimethoprim resistance (dfr)	negative

Virulences

description	result
integrases (intI)	negative
plasmid Vi (tviA)	negative
heat-stable enterotoxin (astA)	positive

For Investigational Use Only. Not Intended for Use in Clinical Diagnostics.

**Operator**	---
**Sample ID**	---
**Experiment ID**	S.e.e._Kambole
**Date of Result**	Thu May 31 10:08:12 2012
**Assay Name**	salm-pm1
**Assay ID**	10624
**Well Position**	00 (00-H)
**Software Version**	0.4
**Device**	---
**biotin**	positive
**negative control**	negative
**invA**	positive
**galF**	positive
**manC**	positive

score	spp	serovare	strain	group O			H1	H2
3.1 (98.78 %)	I	Kambole	CDC1863	C1 (O:7)	7	6,7	d	1,[2],7
10.36 (95.94 %)	I	Typhisuis	SGSC2527	C1 (O:7)	7	6,7	c	1,5

Resistance Genotyping

description	result
aminoglycoside resistance (aac, aad, ant, arm)	negative
beta lactam resistance (dha, ctxM, pse, aac, blaCNY, oxa, cmy, per, shv, tem)	negative
bleomycin resistance (ble)	negative
chloramphenicol resistance (cat, cml)	negative
erythomycin and roxythromycin resistance (qnr)	negative
erythromycin resistance (ere)	negative
florfenicol and chloramphenicol resistance (floR)	negative
fluoroquinolone resistance (qnr)	negative
gentamicin resistance (aac)	negative
imipenem resistance (kpc)	negative
kanamycin resistance (aph)	negative
quinolone resistance (qnr)	negative
streptomycin resistance (sph, Str)	negative
sulfonamide resistance (sul)	negative
tetracycline resistance (tet)	negative
trimethoprim resistance (dfr)	negative

Virulences

description	result
heat-stable enterotoxin (astA)	negative
integrases (intI)	negative
plasmid Vi (tviA)	negative

For Investigational Use Only. Not Intended for Use in Clinical Diagnostics.

**Operator**	---
**Sample ID**	---
**Experiment ID**	S.e.e._Montevideo
**Date of Result**	Thu May 31 10:08:18 2012
**Assay Name**	salm-pm1
**Assay ID**	10624
**Well Position**	00 (00-H)
**Software Version**	0.4
**Device**	---
**biotin**	positive
**negative control**	negative
**invA**	positive
**galF**	positive
**manC**	positive

score	spp	serovare	strain	group O			H1	H2
1.22 (99.52 %)	I	Montevideo	CDC1904	C1 (O:7)	7	6,7,14	g,m,[p],s	[1,2,7]
3.33 (98.7 %)	I	Montevideo	SA20030523	C1 (O:7)	7	6,7,14	g,m,[p],s	[1,2,7]

Resistance Genotyping

description	result
aminoglycoside resistance (aac, aad, ant, arm)	negative
beta lactam resistance (dha, ctxM, pse, aac, blaCNY, oxa, cmy, per, shv, tem)	negative
bleomycin resistance (ble)	negative
chloramphenicol resistance (cat, cml)	negative
erythomycin and roxythromycin resistance (qnr)	negative
erythromycin resistance (ere)	negative
florfenicol and chloramphenicol resistance (floR)	negative
fluoroquinolone resistance (qnr)	negative
gentamicin resistance (aac)	negative
imipenem resistance (kpc)	negative
kanamycin resistance (aph)	negative
quinolone resistance (qnr)	negative
streptomycin resistance (sph, Str)	negative
sulfonamide resistance (sul)	negative
tetracycline resistance (tet)	negative
trimethoprim resistance (dfr)	negative

Virulences

description	result
heat-stable enterotoxin (astA)	negative
integrases (intI)	negative
plasmid Vi (tviA)	negative

For Investigational Use Only. Not Intended for Use in Clinical Diagnostics.

**Operator**	---
**Sample ID**	---
**Experiment ID**	S.e.e._Brandenburg
**Date of Result**	Thu May 31 18:20:06 2012
**Assay Name**	salm-pm1
**Assay ID**	10624
**Well Position**	00 (00-H)
**Software Version**	0.4
**Device**	---
**biotin**	positive
**negative control**	negative
**invA**	positive
**galF**	positive
**manC**	positive

score	spp	serovare	strain	group O			H1	H2
0.0 (100.0 %)	I	Brandenburg	CDC2519	B (O:4)	4	4,[5],12	l,v	e,n,z15
7.46 (97.07 %)	I	Sandiego	CDC18	B (O:4)	4	1,4,[5],12	e,h	e,n,z15

Resistance Genotyping

description	result
aminoglycoside resistance (aac, aad, ant, arm)	negative
beta lactam resistance (dha, ctxM, pse, aac, blaCNY, oxa, cmy, per, shv, tem)	negative
bleomycin resistance (ble)	negative
chloramphenicol resistance (cat, cml)	negative
erythomycin and roxythromycin resistance (qnr)	negative
erythromycin resistance (ere)	negative
florfenicol and chloramphenicol resistance (floR)	negative
fluoroquinolone resistance (qnr)	negative
gentamicin resistance (aac)	negative
imipenem resistance (kpc)	negative
kanamycin resistance (aph)	negative
quinolone resistance (qnr)	negative
streptomycin resistance (sph, Str)	positive
sulfonamide resistance (sul)	negative
tetracycline resistance (tet)	positive
trimethoprim resistance (dfr)	negative

Virulences

description	result
heat-stable enterotoxin (astA)	negative
integrases (intI)	negative
plasmid Vi (tviA)	negative

For Investigational Use Only. Not Intended for Use in Clinical Diagnostics.

**Operator**	---
**Sample ID**	---
**Experiment ID**	S.e.e. Franken
**Date of Result**	Thu May 31 09:53:48 2012
**Assay Name**	salm-pm1
**Assay ID**	10624
**Well Position**	00 (00-H)
**Software Version**	0.4
**Device**	---
**biotin**	positive
**negative control**	negative
**invA**	positive
**galF**	positive
**manC**	positive

score	spp	serovare	strain	group O			H1	H2
0.73 (99.72 %)	I	Franken	CDC2570	D1 (O:9)	9	9,12	z6	z67
7.43 (97.08 %)	I	Fresno	CDC1412	D2 (O:9,46)	9,46	9,46	z38	-

Resistance Genotyping

description	result
aminoglycoside resistance (aac, aad, ant, arm)	negative
beta lactam resistance (dha, ctxM, pse, aac, blaCNY, oxa, cmy, per, shv, tem)	negative
bleomycin resistance (ble)	negative
chloramphenicol resistance (cat, cml)	negative
erythomycin and roxythromycin resistance (qnr)	negative
erythromycin resistance (ere)	negative
florfenicol and chloramphenicol resistance (floR)	negative
fluoroquinolone resistance (qnr)	negative
gentamicin resistance (aac)	negative
imipenem resistance (kpc)	negative
kanamycin resistance (aph)	negative
quinolone resistance (qnr)	negative
streptomycin resistance (sph, Str)	negative
sulfonamide resistance (sul)	negative
tetracycline resistance (tet)	negative
trimethoprim resistance (dfr)	negative

Virulences

description	result
heat-stable enterotoxin (astA)	negative
integrases (intI)	negative
plasmid Vi (tviA)	negative

For Investigational Use Only. Not Intended for Use in Clinical Diagnostics.

**Operator**	---
**Sample ID**	---
**Experiment ID**	S.e.e._Singapore
**Date of Result**	Thu May 31 10:07:39 2012
**Assay Name**	salm-pm1
**Assay ID**	10624
**Well Position**	00 (00-H)
**Software Version**	0.4
**Device**	---
**biotin**	positive
**negative control**	negative
**invA**	positive
**galF**	positive
**manC**	positive

score	spp	serovare	strain	group O			H1	H2
0.6 (99.77 %)	I	Singapore	CDC010011	C1 (O:7)	7	6,7	k	e,n,x
11.11 (95.64 %)	I	Kenya	CDC497	C1 (O:7)	7	6,7	l,z13	e,n,x

Resistance Genotyping

description	result
aminoglycoside resistance (aac, aad, ant, arm)	positive
beta lactam resistance (dha, ctxM, pse, aac, blaCNY, oxa, cmy, per, shv, tem)	negative
bleomycin resistance (ble)	negative
chloramphenicol resistance (cat, cml)	negative
erythomycin and roxythromycin resistance (qnr)	negative
erythromycin resistance (ere)	negative
florfenicol and chloramphenicol resistance (floR)	negative
fluoroquinolone resistance (qnr)	negative
gentamicin resistance (aac)	negative
imipenem resistance (kpc)	negative
kanamycin resistance (aph)	negative
quinolone resistance (qnr)	negative
streptomycin resistance (sph, Str)	positive
sulfonamide resistance (sul)	positive
tetracycline resistance (tet)	positive
trimethoprim resistance (dfr)	positive

Virulences

description	result
heat-stable enterotoxin (astA)	negative
plasmid Vi (tviA)	negative
integrases (intI)	positive

For Investigational Use Only. Not Intended for Use in Clinical Diagnostics.

**Operator**	---
**Sample ID**	---
**Experiment ID**	S.e.e._Heidelberg
**Date of Result**	Thu May 31 09:55:11 2012
**Assay Name**	salm-pm1
**Assay ID**	10624
**Well Position**	00 (00-H)
**Software Version**	0.4
**Device**	---
**biotin**	positive
**negative control**	negative
**invA**	positive
**galF**	positive
**manC**	positive

score	spp	serovare	strain	group O			H1	H2
1.25 (99.51 %)	I	Heidelberg	CDC16	B (O:4)	4	1,4,[5],12	r	1,2
2.82 (98.89 %)	I	Heidelberg	DSM9379	B (O:4)	4	1,4,[5],12	r	1,2

Resistance Genotyping

description	result
aminoglycoside resistance (aac, aad, ant, arm)	negative
beta lactam resistance (dha, ctxM, pse, aac, blaCNY, oxa, cmy, per, shv, tem)	negative
bleomycin resistance (ble)	negative
chloramphenicol resistance (cat, cml)	negative
erythomycin and roxythromycin resistance (qnr)	negative
erythromycin resistance (ere)	negative
florfenicol and chloramphenicol resistance (floR)	negative
fluoroquinolone resistance (qnr)	negative
gentamicin resistance (aac)	negative
imipenem resistance (kpc)	negative
kanamycin resistance (aph)	negative
quinolone resistance (qnr)	negative
streptomycin resistance (sph, Str)	negative
sulfonamide resistance (sul)	negative
tetracycline resistance (tet)	negative
trimethoprim resistance (dfr)	negative

Virulences

description	result
heat-stable enterotoxin (astA)	negative
integrases (intI)	negative
plasmid Vi (tviA)	negative

For Investigational Use Only. Not Intended for Use in Clinical Diagnostics.

**Operator**	---
**Sample ID**	---
**Experiment ID**	Salmonella enterica ssp. enterica_Gallinarum
**Date of Result**	Thu May 31 18:07:58 2012
**Assay Name**	salm-pm1
**Assay ID**	10624
**Well Position**	00 (00-H)
**Software Version**	0.4
**Device**	---
**biotin**	positive
**negative control**	negative
**invA**	positive
**galF**	positive
**manC**	positive

score	spp	serovare	strain	group O			H1	H2
0.0 (100.0 %)	I	Gallinarum	DSM13674	D1 (O:9)	9	1,9,12	-	-
2.95 (98.84 %)	I	Gallinarum	CDC74	D1 (O:9)	9	1,9,12	-	-

Resistance Genotyping

description	result
aminoglycoside resistance (aac, aad, ant, arm)	negative
beta lactam resistance (dha, ctxM, pse, aac, blaCNY, oxa, cmy, per, shv, tem)	negative
bleomycin resistance (ble)	negative
chloramphenicol resistance (cat, cml)	negative
erythomycin and roxythromycin resistance (qnr)	negative
erythromycin resistance (ere)	negative
florfenicol and chloramphenicol resistance (floR)	negative
fluoroquinolone resistance (qnr)	negative
gentamicin resistance (aac)	negative
imipenem resistance (kpc)	negative
kanamycin resistance (aph)	negative
quinolone resistance (qnr)	negative
streptomycin resistance (sph, Str)	negative
sulfonamide resistance (sul)	negative
tetracycline resistance (tet)	negative
trimethoprim resistance (dfr)	negative

Virulences

description	result
heat-stable enterotoxin (astA)	negative
integrases (intI)	negative
plasmid Vi (tviA)	negative

For Investigational Use Only. Not Intended for Use in Clinical Diagnostics.

**Operator**	---
**Sample ID**	---
**Experiment ID**	Salmonella enterica ssp. enterica_Typhimurium (LT2)
**Date of Result**	Thu May 31 10:00:35 2012
**Assay Name**	salm-pm1
**Assay ID**	10624
**Well Position**	00 (00-H)
**Software Version**	0.4
**Device**	---
**biotin**	positive
**negative control**	negative
**invA**	positive
**galF**	positive
**manC**	positive

score	spp	serovare	strain	group O			H1	H2
5.22 (97.95 %)	I	Typhimurium	DSM17058	B (O:4)	4	1,4,[5],12	i	1,2
5.97 (97.66 %)	I	Typhimurium	DSM19587	B (O:4)	4	1,4,[5],12	i	1,2

Resistance Genotyping

description	result
aminoglycoside resistance (aac, aad, ant, arm)	negative
beta lactam resistance (dha, ctxM, pse, aac, blaCNY, oxa, cmy, per, shv, tem)	negative
bleomycin resistance (ble)	negative
chloramphenicol resistance (cat, cml)	negative
erythomycin and roxythromycin resistance (qnr)	negative
erythromycin resistance (ere)	negative
florfenicol and chloramphenicol resistance (floR)	negative
fluoroquinolone resistance (qnr)	negative
gentamicin resistance (aac)	negative
imipenem resistance (kpc)	negative
kanamycin resistance (aph)	negative
quinolone resistance (qnr)	negative
streptomycin resistance (sph, Str)	negative
sulfonamide resistance (sul)	negative
tetracycline resistance (tet)	negative
trimethoprim resistance (dfr)	negative

Virulences

description	result
heat-stable enterotoxin (astA)	negative
integrases (intI)	negative
plasmid Vi (tviA)	negative

For Investigational Use Only. Not Intended for Use in Clinical Diagnostics.

Operator ---
Sample ID ---
Experiment ID Salmonella enterica spp. enterica_Enteritidis
Date of Result Thu May 31 18:12:09 2012
Assay Name salm-pm1
Assay ID 10624
Well Position 00 (00-H)
Software Version 0.4
Device ---
biotin positive
negative control negative
invA positive
galF positive
manC positive

score	spp	serovare	strain	group O			H1	H2
0.0 (100.0 %)	I	Enteritidis	ST010	D1 (O:9)	9	1,9,12	g,m	-
2.08 (99.18 %)	I	Enteritidis	DSM17420	D1 (O:9)	9	1,9,12	g,m	-

Resistance Genotyping

description	result
aminoglycoside resistance (aac, aad, ant, arm)	negative
beta lactam resistance (dha, ctxM, pse, aac, blaCNY, oxa, cmy, per, shv, tem)	negative
bleomycin resistance (ble)	negative
chloramphenicol resistance (cat, cml)	negative
erythomycin and roxythromycin resistance (qnr)	negative
erythromycin resistance (ere)	negative
florfenicol and chloramphenicol resistance (floR)	negative
fluoroquinolone resistance (qnr)	negative
gentamicin resistance (aac)	negative
imipenem resistance (kpc)	negative
kanamycin resistance (aph)	negative
quinolone resistance (qnr)	negative
streptomycin resistance (sph, Str)	negative
sulfonamide resistance (sul)	negative
tetracycline resistance (tet)	negative
trimethoprim resistance (dfr)	negative

Virulences

description	result
heat-stable enterotoxin (astA)	negative
integrases (intI)	negative
plasmid Vi (tviA)	negative

For Investigational Use Only. Not Intended for Use in Clinical Diagnostics.

**Operator**	---
**Sample ID**	---
**Experiment ID**	Salmonella enterica ssp. enterica
**Date of Result**	Mon Nov 28 17:42:10 2011
**Assay Name**	salm-pm1
**Assay ID**	10624
**Well Position**	00 (00-H)
**Software Version**	0.4
**Device**	---
**biotin**	positive
**negative control**	negative

score	spp	species	strain	stringenz	serovare	group O			H1	H2
99.78 %	I	Salmonella enterica ssp. enterica	DSM4224		Abony	1,4, [5],12,27			b	e,n,x
97.05 %	I	Salmonella enterica spp. enterica	SGSC2460		Brandenburg	4			l,v	e,n,z15

Resistance Genotyping

description	result
aminoglycoside resistance (aac, aad, ant, arm)	negative
beta lactam resistance (dha, ctxM, pse, aac, blaCNY, oxa, cmy, per, shv, tem)	negative
bleomycin resistance (ble)	negative
chloramphenicol resistance (cat, cml)	negative
erythomycin and roxythromycin resistance (qnr)	negative
erythromycin resistance (ere)	negative
florfenicol and chloramphenicol resistance (floR)	negative
fluoroquinolone resistance (qnr)	negative
gentamicin resistance (aac)	negative
imipenem resistance (kpc)	negative
kanamycin resistance (aph)	negative
located next to mphA, function unknown (mrx)	negative
quinolone resistance (qnr)	negative
streptomycin resistance (sph, Str)	negative
sulfonamide resistance (sul)	negative
tetracycline resistance (tet)	negative
trimethoprim resistance (dfr)	negative

Virulances

description	result
enteroxin (astA)	negative
integrases (intI)	negative
plasmid Vi (tviA)	negative

For Investigational Use Only. Not Intended for Use in Clinical Diagnostics.

**Operator**	---
**Sample ID**	---
**Experiment ID**	Salmonella bongori
**Date of Result**	Thu May 31 18:10:57 2012
**Assay Name**	salm-pm1
**Assay ID**	10624
**Well Position**	00 (00-H)
**Software Version**	0.4
**Device**	---
**biotin**	positive
**negative control**	negative
**invA**	positive
**galF**	negative
**manC**	negative

score	spp	serovare	strain	group O			H1	H2
0.0 (100.0 %)	V	66:z41:-	DSM13774	O:66	66	66	z41	-
11.08 (95.65 %)	I	Alachua	CDC325	O (O:35)	35	35	z4,z23	-

Resistance Genotyping

description	result
aminoglycoside resistance (aac, aad, ant, arm)	negative
beta lactam resistance (dha, ctxM, pse, aac, blaCNY, oxa, cmy, per, shv, tem)	negative
bleomycin resistance (ble)	negative
chloramphenicol resistance (cat, cml)	negative
erythomycin and roxythromycin resistance (qnr)	negative
erythromycin resistance (ere)	negative
florfenicol and chloramphenicol resistance (floR)	negative
fluoroquinolone resistance (qnr)	negative
gentamicin resistance (aac)	negative
imipenem resistance (kpc)	negative
kanamycin resistance (aph)	negative
quinolone resistance (qnr)	negative
streptomycin resistance (sph, Str)	negative
sulfonamide resistance (sul)	negative
tetracycline resistance (tet)	negative
trimethoprim resistance (dfr)	negative

Virulences

description	result
heat-stable enterotoxin (astA)	negative
integrases (intI)	negative
plasmid Vi (tviA)	negative

For Investigational Use Only. Not Intended for Use in Clinical Diagnostics.

**Operator**	---
**Sample ID**	---
**Experiment ID**	Salmonella enterica ssp. enterica_Paratyphi B
**Date of Result**	Thu May 31 09:55:05 2012
**Assay Name**	salm-pm1
**Assay ID**	10624
**Well Position**	00 (00-H)
**Software Version**	0.4
**Device**	---
**biotin**	positive
**negative control**	negative
**invA**	positive
**galF**	positive
**manC**	positive

score	spp	serovare	strain	group O			H1	H2
0.61 (99.76 %)	I	Paratyphi B	10-62165	B (O:4)	4	1,4,[5],12	b	1,2
4.91 (98.07 %)	I	Heidelberg	DSM9379	B (O:4)	4	1,4,[5],12	r	1,2

Resistance Genotyping

description	result
aminoglycoside resistance (aac, aad, ant, arm)	negative
beta lactam resistance (dha, ctxM, pse, aac, blaCNY, oxa, cmy, per, shv, tem)	negative
bleomycin resistance (ble)	negative
chloramphenicol resistance (cat, cml)	negative
erythomycin and roxythromycin resistance (qnr)	negative
erythromycin resistance (ere)	negative
florfenicol and chloramphenicol resistance (floR)	negative
fluoroquinolone resistance (qnr)	negative
gentamicin resistance (aac)	negative
imipenem resistance (kpc)	negative
kanamycin resistance (aph)	negative
quinolone resistance (qnr)	negative
streptomycin resistance (sph, Str)	negative
sulfonamide resistance (sul)	negative
tetracycline resistance (tet)	negative
trimethoprim resistance (dfr)	negative

Virulences

description	result
heat-stable enterotoxin (astA)	negative
integrases (intI)	negative
plasmid Vi (tviA)	negative

For Investigational Use Only. Not Intended for Use in Clinical Diagnostics.

**Operator**	---
**Sample ID**	---
**Experiment ID**	Salmonella enterica spp. enterica_Bredeney
**Date of Result**	Mon Nov 28 17:39:03 2011
**Assay Name**	salm-pm1
**Assay ID**	10624
**Well Position**	00 (00-H)
**Software Version**	0.4
**Device**	---
**biotin**	positive
**negative control**	negative

score	spp	species	strain	stringenz	serovare	group O			H1	H2
97.43 %	I	Salmonella enterica spp. enterica	96		Bredeney	4			l,v	1,7
95.64 %	I	Salmonella enterica spp. enterica	SK025		Give	3,10			l,v	1,7

Resistance Genotyping

description	result
aminoglycoside resistance (aac, aad, ant, arm)	negative
beta lactam resistance (dha, ctxM, pse, aac, blaCNY, oxa, cmy, per, shv, tem)	negative
bleomycin resistance (ble)	negative
chloramphenicol resistance (cat, cml)	negative
erythomycin and roxythromycin resistance (qnr)	negative
erythromycin resistance (ere)	negative
florfenicol and chloramphenicol resistance (floR)	negative
fluoroquinolone resistance (qnr)	negative
gentamicin resistance (aac)	negative
imipenem resistance (kpc)	negative
kanamycin resistance (aph)	negative
located next to mphA, function unknown (mrx)	negative
quinolone resistance (qnr)	negative
streptomycin resistance (sph, Str)	negative
sulfonamide resistance (sul)	negative
tetracycline resistance (tet)	positive
trimethoprim resistance (dfr)	positive

Virulances

description	result
enteroxin (astA)	negative
integrases (intI)	negative
plasmid Vi (tviA)	negative

For Investigational Use Only. Not Intended for Use in Clinical Diagnostics.

**Operator**	---
**Sample ID**	---
**Experiment ID**	Salmonella enterica spp. enterica_Panama
**Date of Result**	Thu May 31 18:13:51 2012
**Assay Name**	salm-pm1
**Assay ID**	10624
**Well Position**	00 (00-H)
**Software Version**	0.4
**Device**	---
**biotin**	positive
**negative control**	negative
**invA**	positive
**galF**	positive
**manC**	positive

score	spp	serovare	strain	group O			H1	H2
0.0 (100.0 %)	I	Panama	SGSC2496	D1 (O:9)	9	1,9,12	l,v	1,5
1.91 (99.25 %)	I	Panama	K017	D1 (O:9)	9	1,9,12	l,v	1,5

Resistance Genotyping

description	result
aminoglycoside resistance (aac, aad, ant, arm)	negative
beta lactam resistance (dha, ctxM, pse, aac, blaCNY, oxa, cmy, per, shv, tem)	negative
bleomycin resistance (ble)	negative
chloramphenicol resistance (cat, cml)	negative
erythomycin and roxythromycin resistance (qnr)	negative
erythromycin resistance (ere)	negative
florfenicol and chloramphenicol resistance (floR)	negative
fluoroquinolone resistance (qnr)	negative
gentamicin resistance (aac)	negative
imipenem resistance (kpc)	negative
kanamycin resistance (aph)	negative
quinolone resistance (qnr)	negative
streptomycin resistance (sph, Str)	negative
sulfonamide resistance (sul)	negative
tetracycline resistance (tet)	negative
trimethoprim resistance (dfr)	negative

Virulences

description	result
heat-stable enterotoxin (astA)	negative
integrases (intI)	negative
plasmid Vi (tviA)	negative
