# Supplementary material for: A Proof-of-Concept Protein Microarray-Based Approach for Serotyping of Salmonella enterica Strains
Source: Pathogens. 2024 Apr 25;13(5):355. doi: 10.3390/pathogens13050355 (PMC11124431; doi:10.3390/pathogens13050355)
Supplement: Supplementary file 1 [file pathogens-13-00355-s001.zip › Supplementary Table S2.pdf]

Supplementary Table S2: *All spotted polyclonal antisera and their dilutions are listed. The numbers represent positions on the microarray (see Supplemental Table S3).*

Well	Number	Substance name	Dilution
A1	1	TR1101_Anti-Salm_A-67_Vi_1_6_D12	1:6
B1	2	TR1101_Anti-Salm_A-67_Vi_1_10_D12	1:10
C1	3	TR1101_Anti-Salm_A-67_Vi_1_6	1:6
D1	4	TR1101_Anti-Salm_A-67_Vi_1_8	1:8
E1	5	TR1101_Anti-Salm_A-67_Vi_1_10	1:10
F1	6	TR1101_Anti-Salm_A-67_Vi_1_20	1:20
G1	7	TR1111_Anti-Salm_I_A-E_Vi_1_6_D12	1:6
H1	8	TR1111_Anti-Salm_I_A-E_Vi_1_10_D12	1:10
I1	9	TR1111_Anti-Salm_I_A-E_Vi_1_6	1:6
J1	10	TR1111_Anti-Salm_I_A-E_Vi_1_8	1:8
K1	11	TR1111_Anti-Salm_I_A-E_Vi_1_10	1:10
L1	12	TR1111_Anti-Salm_I_A-E_Vi_1_20	1:20
M1	13	TR1307_Anti-Salm_O-9_1_6_D12	1:6
N1	14	TR1307_Anti-Salm_O-9_1_10_D12	1:10
O1	15	TR1307_Anti-Salm_O-9_1_6	1:6
P1	16	TR1307_Anti-Salm_O-9_1_8	1:8
A2	17	TR1307_Anti-Salm_O-9_1_10	1:10
B2	18	TR1307_Anti-Salm_O-9_1_20	1:20
C2	19	TR1302_Anti-Salm_O-4_1_6_D12	1:6
D2	20	TR1302_Anti-Salm_O-4_1_10_D12	1:10
E2	21	TR1302_Anti-Salm_O-4_1_6	1:6
F2	22	TR1302_Anti-Salm_O-4_1_8	1:8
G2	23	TR1302_Anti-Salm_O-4_1_10	1:10
H2	24	TR1302_Anti-Salm_O-4_1_20	1:20
I2	25	TR1303_Anti-Salm_O-5_1_6_D12	1:6
J2	26	TR1303_Anti-Salm_O-5_1_10_D12	1:10
K2	27	TR1303_Anti-Salm_O-5_1_6	1:6
L2	28	TR1303_Anti-Salm_O-5_1_8	1:8
M2	29	TR1303_Anti-Salm_O-5_1_10	1:10
N2	30	TR1303_Anti-Salm_O-5_1_20	1:20
O2	31	TR1437_Anti-Salm_H-1_1_6_D12	1:6
P2	32	TR1437_Anti-Salm_H-1_1_10_D12	1:10
A3	33	TR1437_Anti-Salm_H-1_1_6	1:6
B3	34	TR1437_Anti-Salm_H-1_1_8	1:8
C3	35	TR1437_Anti-Salm_H-1_1_10	1:10
D3	36	TR1437_Anti-Salm_H-1_1_20	1:20
E3	37	TR1433_Anti-Salm_H-2_1_6_D12	1:6
F3	38	TR1433_Anti-Salm_H-2_1_10_D12	1:10
G3	39	TR1433_Anti-Salm_H-2_1_6	1:6
H3	40	TR1433_Anti-Salm_H-2_1_8	1:8
I3	41	TR1433_Anti-Salm_H-2_1_10	1:10
J3	42	TR1433_Anti-Salm_H-2_1_20	1:20
K3	43	TR1410_Anti-Salm_H-i_1_6_D12	1:6
L3	44	TR1410_Anti-Salm_H-i_1_10_D12	1:10
M3	45	TR1410_Anti-Salm_H-i_1_6	1:6
N3	46	TR1410_Anti-Salm_H-i_1_8	1:8
O3	47	TR1410_Anti-Salm_H-i_1_10	1:10
P3	48	TR1410_Anti-Salm_H-i_1_20	1:20
A4	49	TR1406_Anti-Salm_H-g_1_6_D12	1:6
B4	50	TR1406_Anti-Salm_H-g_1_10_D12	1:10

Well	Number	Substance name	Dilution
C4	51	TR1406_Anti-Salm_H-g_1_6	1:6
D4	52	TR1406_Anti-Salm_H-g_1_8	1:8
E4	53	TR1406_Anti-Salm_H-g_1_10	1:10
F4	54	TR1406_Anti-Salm_H-g_1_20	1:20
G4	55	TS1413_Anti-Salm_H-m_1_6_D12	1:6
H4	56	TS1413_Anti-Salm_H-m_1_4_D12	1:10
I4	57	TS1413_Anti-Salm_H-m_1_6	1:6
J4	58	TS1413_Anti-Salm_H-m_1_8	1:8
K4	59	TS1413_Anti-Salm_H-m_1_10	1:10
L4	60	TS1413_Anti-Salm_H-m_1_20	1:20
M4	61	Buffer	
N4	62	Protein-A/G-HRP_0.2	spot-conc. 0.2
