# Supplementary material for: A Proof-of-Concept Protein Microarray-Based Approach for Serotyping of Salmonella enterica Strains
Source: Pathogens. 2024 Apr 25;13(5):355. doi: 10.3390/pathogens13050355 (PMC11124431; doi:10.3390/pathogens13050355)
Supplement: Supplementary file 1 [file pathogens-13-00355-s001.zip › Supplementary Table S3.pdf]

Supplementary Table S3: *Spotting grid of all antisera. Spotted using a M2 Spotter. The numbers represent the antisera on the microarray (see Supplemental Table S2). Number 62 are postive controls, but also used as orientations spots for the reader software.*

**62**	46	47	48	49	50	51	52	53	54	55	56	57	58	59	60	**62**
	31	32	33	34	35	36	37	38	39	40	41	42	43	44	45	
**62**	16	17	18	19	20	21	22	23	24	25	26	27	28	29	30	**62**
**62**	1	2	3	4	5	6	7	8	9	10	11	12	13	14	15	**62**
	46	47	48	49	50	51	52	53	54	55	56	57	58	59	60	**62**
	31	32	33	34	35	36	37	38	39	40	41	42	43	44	45	**62**
	16	17	18	19	20	21	22	23	24	25	26	27	28	29	30	**62**
	1	2	3	4	5	6	7	8	9	10	11	12	13	14	15	**62**
	61				61				61				61			
	46	47	48	49	50	51	52	53	54	55	56	57	58	59	60	
	31	32	33	34	35	36	37	38	39	40	41	42	43	44	45	
	16	17	18	19	20	21	22	23	24	25	26	27	28	29	30	
	1	2	3	4	5	6	7	8	9	10	11	12	13	14	15	
	46	47	48	49	50	51	52	53	54	55	56	57	58	59	60	
	31	32	33	34	35	36	37	38	39	40	41	42	43	44	45	
	16	17	18	19	20	21	22	23	24	25	26	27	28	29	30	
**62**	1	2	3	4	5	6	7	8	9	10	11	12	13	14	15	**62**
